# Weather warning archives reveal spatio-temporal hot spots of compound natural hazards

**DOI:** 10.1038/s41598-025-96842-6

**Published:** 2025-04-17

**Authors:** Wei Yang, Jonas Olsson, Peter Berg, Lennart Simonsson

**Affiliations:** 1 Hydrology Research, Swedish Meteorological and Hydrological Institute, Norrköping, Sweden; 2 Division of Water Resources Engineering, Lund University, Lund, Sweden

**Keywords:** Hydrology, Natural hazards

## Abstract

Individual natural hazards may be combined in different ways, leading to cascading or co-occurring effects, turning them into compound hazards. However, assessment of individual as well as compound hazards is often hampered by short or incomplete observational records of actual hazards, and records of various hazards that do not easily combine. In this study we propose an alternative way to detect potential risk of compound natural hazards via archived severe weather warnings. We investigate weather warnings in Sweden from 2011 to 2020 regarding their distributions and frequencies in time (at daily level) and space (at warning district level) from both an individual and compound perspective. We illustrate the methodology and results by focusing on compound flood-related risk, generated by combinations of heavy rainfall, high streamflow and high sea level, and contextualize with two actual compound flood events in Sweden. We find compound fluvial and coastal flood risk primarily along the southwest coast during the winter half year as well as compound fluvio-pluvial flood risk during the summer half year. The results show that severe weather warnings can be used to assess the frequency and compounding nature of natural hazards, as well as to identify actual cases for further investigation, and we encourage similar investigations elsewhere.

**Supplementary Information:**

The online version contains supplementary material available at 10.1038/s41598-025-96842-6.

## Introduction

The interaction of two or more natural hazards may lead to an impact that exceeds the sum of the individual impacts^1,2^. Such an event is generally termed either multi-hazard or compound event, where compound event is frequently used in the weather and climate field. The term includes temporal sequences of events or compound events involving simultaneous combinations of several hazards, each inessentially an extreme by itself, which together constitute an extreme impact^3^.

Several approaches have been made to analyze compound hazards concerning drivers, mechanisms, interactions and impacts through literature reviews, real events and workshops^4–6^. This work has led to typologies or classifications^7,8^ in order to provide a unified basis for investigating compound effects and discussing follow-up guidances. Actual events have been investigated to deepen the understanding of their underlying mechanisms and to identify corresponding drivers for specific conditions, covering a variety of interconnections between natural hazards. Examples include storm surge and precipitation in the Netherlands^9^ and storm surge and high river runoff in Italy^10^, where storylines were used to describe how and why the compound events happened using model simulations and statistical approaches.

Concerning Sweden, 12 natural hazards (geophysical mass movements, storm, heavy precipitation, lightning, heat wave, cold spell, flood, drought, hydrological mass movements, avalanche, wildfires, pest infestations) are considered relevant in the systematic literature study by Gustafsson et al.^11^. They are characterized by affected areas ranging from local to national scale and a pronounced seasonality, which reflect the meteorological and geographical conditions of Sweden^12^. Examples of recent major events in Sweden include urban pluvial floods in the cities Malmö in 2014^13^ and Gävle in 2021^14^ as well as storms Gudrun in 2005^15,16^ and Hans in 2023^17–20^. Furthermore, wildfires (i.e., grassland fire and forest fire), e.g. in Sala in 2014 and more widespread during summer 2018^21^, have caused severe challenges for rescue services and landowners^22,23^.

Flood hazards (i.e., coastal, pluvial and fluvial flooding) are significant as a type of both primary and secondary hazard^11,24^, i.e. they may not only trigger other hazards (e.g. landslide, debris flow, soil movement, erosion and pest infestation), but they are also caused by other hazards. In Gustafsson et al.^11^, fluvial flooding is identified as the consequence of 11 primary hazards, whereas pluvial flooding was found to be related to heavy rainfall, and its magnitude or frequency of occurrence can be influenced by primary hazards such as heavy rainfall, drought, wildfire and low pressure storms. Coastal flooding was found highly connected to storms whereas no evidence points toward connections amongst the different types of flood hazards, which may be considered a limitation of that study.

Lack of observation data sets of actual natural hazards as well as their associated hydro-meteorological extremes is however a major challenge^25^. Concerning Sweden, no systematic, comprehensive and updated data base with historical natural hazards exists, and further, there are large uncertainties in observation records, with few clear trends caused by limited sampling of events in the monitoring station network. Consequently, it is virtually impossible to meaningfully study compound events (in Sweden) solely based on observations. The main alternative is to use modelling, primarily to generate long periods of temporally and spatially high-resolution realizations of the processes behind different natural hazards^26–29^. In model simulations representing present or future climate, compound events can be identified and analyzed with respect to e.g. frequency and severity. The main advantage includes the production of large data sets, covering (ensembles of) long periods at high resolutions based on state-of-the-art scientific development. The model uncertainty, especially associated with the reproduction of extremes, is the major limitation.

Hydro-meteorological hazards are commonly assessed by operational modelling and forecasting at National Meteorological and Hydrological Services (NMHSs) (in Sweden: Swedish Meteorological and Hydrological Institute, SMHI). Supported by critical thresholds, and (importantly) expert judgement, the NMHSs publicly issue warnings, and other potential notifications, e.g. advisories. These warnings have widely differing durations and cover widely different domains, depending on the hazard (e.g. widespread drought vs. localized cloudburst), but they all have a (more or less) distinct start, end and coverage. Here we hypothesize that historically archived weather warnings (meteorological, hydrological and oceanographical) can be used to quantify the risk of compound events and identify spatial hot spots for different types. An obvious limitation is that the warnings generally do not say anything about the outcome, i.e. whether compound events actually happened or not, although they may be used to identify events that have occurred (see Validation events). The approach however highlights the practical significance of the warnings, which is that the warnings are what society (e.g. administrative county boards and rescue services) has to consider and respond to before the actual outcome is known. To our knowledge, weather warnings have not previously been used for this purpose.

**Fig. 1:**
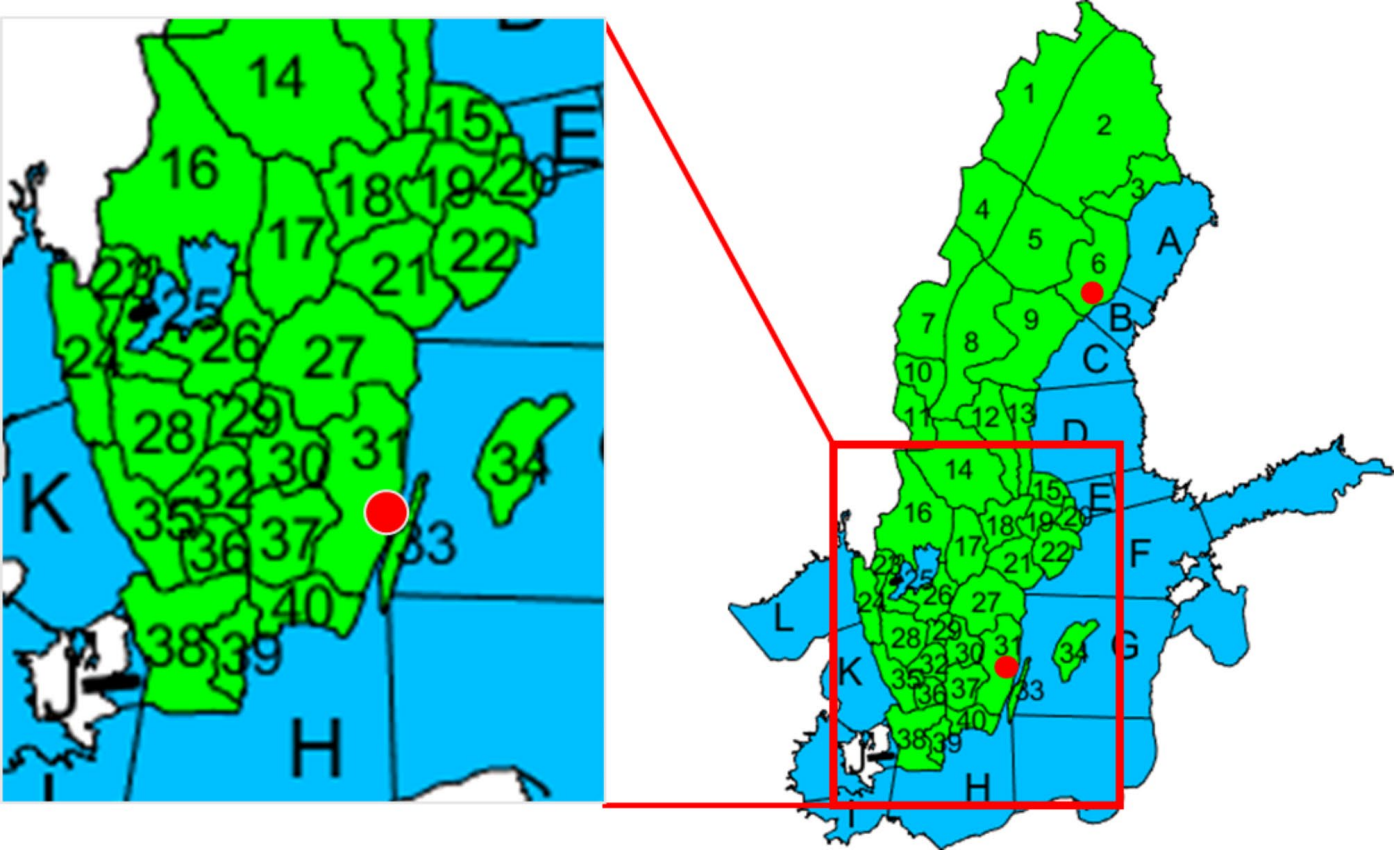
The 40 warning districts on land (in green and numbered) and 12 neighbouring waterbodies (in blue with letters). The names of the warning districts (WD) and waterbodies (WB) are given in the Supplementary Information, Table S1. The red dots show the locations of the two validation events. The maps were generated using the Mapping Toolbox in Matlab version 2018b (https://www.mathworks.com/products/mapping.html).

In light of the above, we analyzed 10 years (2011–2020) of archived weather warnings at SMHI, issued for 40 warning districts (Fig. 1), to primarily identify and quantify the occurrence of multiple co-occurring warnings in a given region. These cases have the potential for generating compound events and require that early responders are on an elevated alert level, compared with single warning cases. Our main objective is to quantify and characterize the spatio-temporal patterns of the three main types of compound flood-related risk – any combinations of heavy rainfall (HR), high streamflow (HQ) and high sea level (HW) – in Sweden. To reach this objective, we develop and apply (*i*) a methodology to convert the original warnings into a suitable format and (*ii*) numerical measures for quantifying the occurrence of multiple warnings.

## Results

### Individual flood-related warnings

Initially, the warnings issued for each single flood-related type, further divided into two or three severity classes (1–3) indicating larger affected areas and/or more serious potential consequences (see Supplementary Information, Table S2), were transformed into binary matrices on day and district levels. Thus, for a certain warning type and a certain combination of day and district, 1 (0) means that this warning was (not) active all or part of this day in all or part of this district. Metrics calculated from these matrices allow quantification of the occurrences of the different warning types in time and space, for individual warnings as well as combinations of warnings (see details in the Methods section). The warnings are thus named by the type of risk (HR, HQ, HW) combined with the severity class (1–3), e.g. HQ2.

**Fig. 2:**
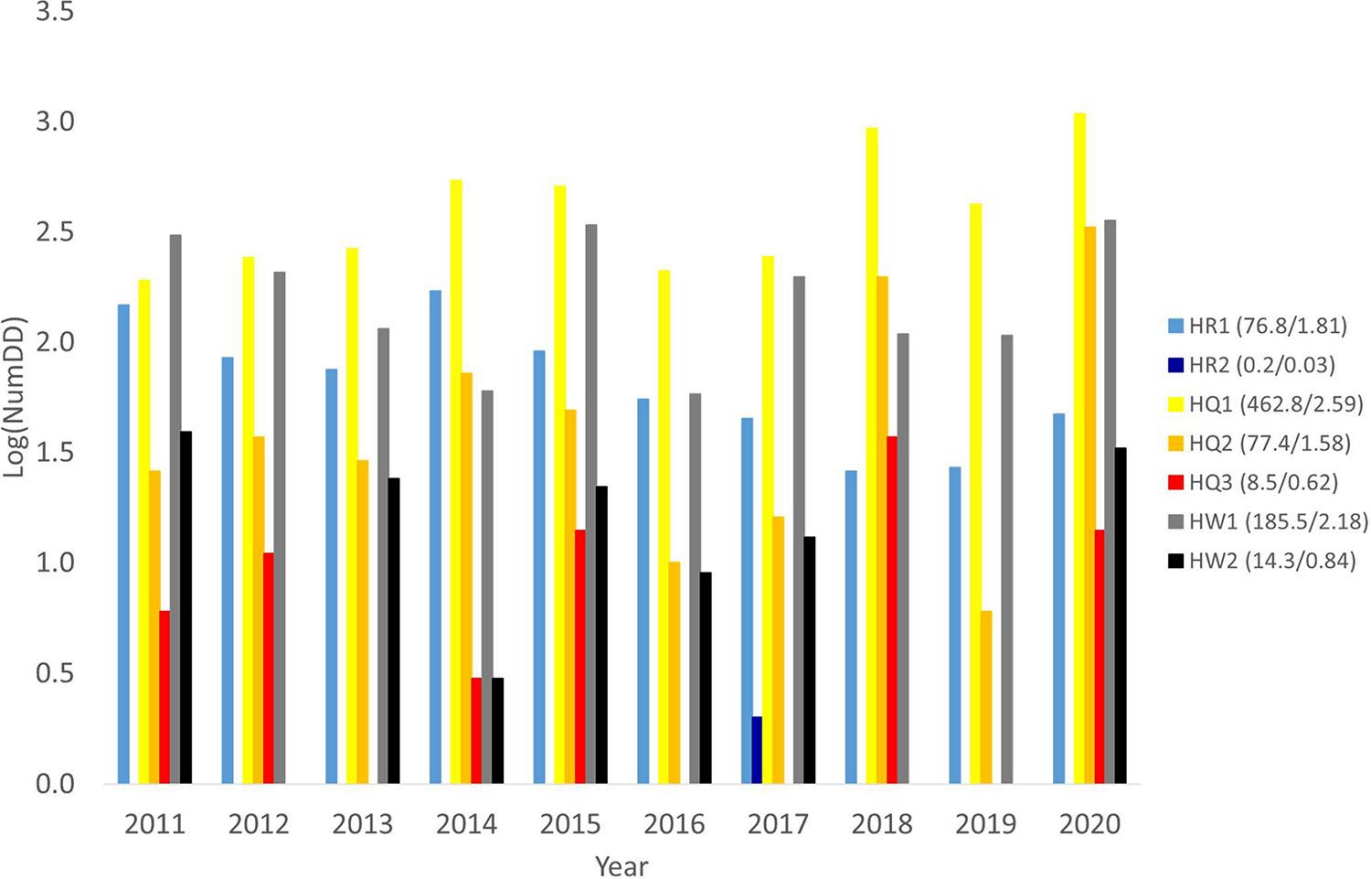
Annual number of issued flood-related warnings (HR = heavy rainfall, HQ = high streamflow and HW = high sea level) from 2011–2020. Note the logarithmic scale on the y-axis, used to clearly visualize the entire range of values. The values in the legend are the 10-year averages (actual value / logarithmic value).

The most basic metric NumDD is a summation over the binary matrices, i.e. reflecting how often a certain warning was issued on any day in any district. Figure 2 shows that the HQ1 is by far the most commonly issued warning type, with almost 500 occurrences (2.59 in logarithm scale) as an annual average, ranging between 190 and 1084 across the individual years. HQ2 occurs every year, on average 77 times, whereas HQ3 is not issued every year and its maximum occurrence reached up to 37 times. HW1 is the second most common warning, with HW2 being rather similar to HQ3 with respect to maximum level and zero years. HR1 is rather similar to HQ2, whereas HR2 is very unusual with only two occurrences in the period. Issued warnings per month on a national basis are shown in the Supplementary Information, Figure S1.

Concerning the interannual variability, we found that in 2015 the country was at enhanced risk of all three types of hazards, as demonstrated by the larger-than-average number of issued warnings for all warning classes. Also 2020 is worthy of notice, with the numbers of HQ2, HQ3, HW1 and HW2 warnings being nearly twice their respective 10-year averages.

Calculations based on the binary matrices reveal various space-time characteristics of the issued warnings (see Supplementary Information, Table S3). For example, an HQ1 warning was issued somewhere in Sweden (i.e. in at least one district) on average 105 days per year (metric: NumDays). This is likely related to the comparatively slow process of (fluvial) flood generation, with warnings often gradually “travelling downstream” together with the peak flow for an extended period of time. The corresponding values for HR1 and HW1 are 18 and 44 days, respectively. The number of districts (out of 40) experiencing a certain type of warning in the 10-year period (metric: NumDistricts) is another characteristic related to risk management. The values of NumDistricts for HR1 and HQ1 are similar and close to 25 districts, although the HQ1 warnings are much more frequently issued. HW warnings affect fewer districts which is expected as they are restricted to coastal areas. Finally, the metric AvgDistricts reveals the number of districts that are affected when a certain type of warning is issued. Class-1 warnings of all types all cover an average of four districts, i.e. they typically extend over a similarly sized domain.

**Fig. 3:**
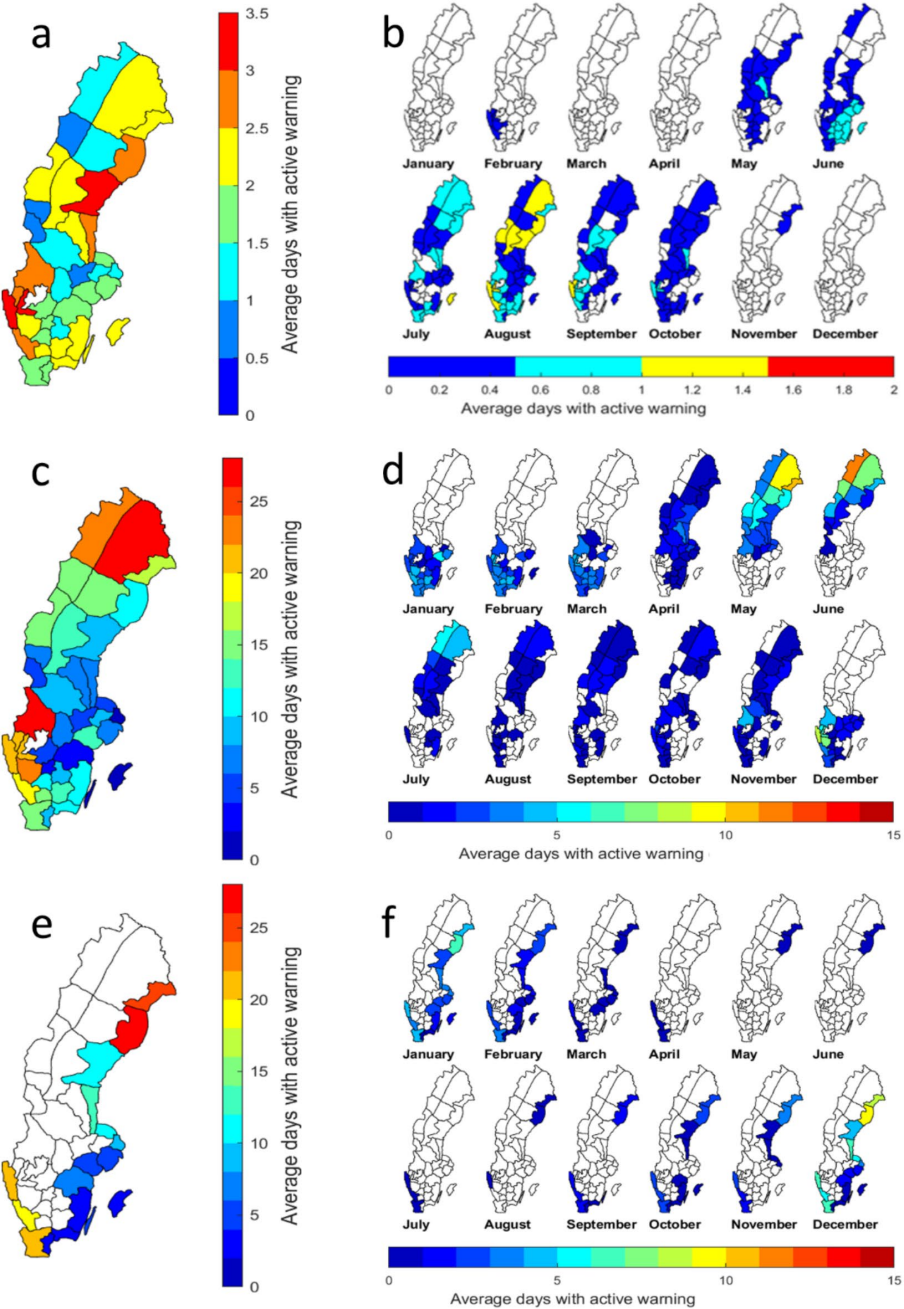
Average number of days per year and per month during 2011–2020 with warnings issued for HR (**a**,** b**), HQ (**c**,** d**) and HW (**e**,** f**). Note the different scales of the colour bars. The maps were generated using the Mapping Toolbox in Matlab version 2018b (https://www.mathworks.com/products/mapping.html).

Figure 3 illustrates the spatio-temporal pattern of the issued single flood-related warnings. As seen from the issued HR warnings, coastal districts, mainly along the northeast and southwest coasts, were most affected (Fig. 3a). This type of risk mainly appeared outside the winter (i.e. snow) season, from May to October (from 0.1 up to 1.4 warning days per month). HR warnings were frequently issued in August, especially in districts in northern Sweden and along the west and the east coasts in southern Sweden (Fig. 3b).

HQ warnings were frequently issued throughout the year with the highest occurrences in Norrbotten (WD2 in Fig. 1) and Värmland (WD16) (red districts in Fig. 3c). They were issued every month but with differences in frequency as well as spatial distribution. From December to March, streamflow warnings were only issued in southern Sweden, mainly in connection with winter storms. From April on, in particular from May to July, the risk spread towards the north as a consequence of rising temperature, increasing snow melt and spring-flood generation (see Fig. 3d).

The highest number of HW warnings was found in the east coastal district of Västerbotten (WD6 in Fig. 1) (Fig. 3e), where the frequency of HW warnings reached its monthly maximum value of more than nine occurrences in December. The second most risk-prone area was found in the northeast coastal districts of Norrbotten (WD3), with 8.1 occurrences in the same month. As elevated sea levels often occur during the winter season, this type of warning was found mainly from November to February (Fig. 3f).

### Combinations of flood-related warnings

Focusing on the potential risk of any combination of the three flood-related hazards in the same district on the same day (i.e., NumDD), the most prevalent combination is identified as HQ and HW (158 occasions). This is the third most common combination of all warnings, exceeded only by the combinations of HW and strong wind gusts (390) and HW and heavy snowfall (249) (Table S4, above diagonal). Also, the combination of HR and HQ is rather common (93) whereas HR and HW warnings rarely co-occur due to different seasonality (Fig. 3). In terms of NumDays, the appearance of HQ risk shows strong co-occurrence with HW (80 days) and relatively strong co-occurrence with HR (39 days). Similar to NumDD, the combination of HQ and HW is the third most common also in terms of NumDays (Table S4, below diagonal). Warnings of HR and HW are seldom issued for the same day (4 days).

**Table 1:** A summary of the co-occurrence of combinations of the three flood-related hazards during 2011–2020.

	HR	HQ	HW
HR	-	93/39	8/4
HQ	23/2.4	-	158/80
HW	4/2.0	9/2.0	-

Above diagonal: NumDD/NumDays. Below diagonal: numdistrics/avgdistricts.

Considering the number of districts affected by a combined hazard on at least one day during the 10-year period (i.e., NumDistricts), the combination of HR and HQ affected 23 districts (i.e. >50% of all districts), (Table 1). The most common combinations in terms of NumDistricts include heavy snowfall, either together with strong wind gusts (31) or HQ (26) (Table S5, above diagonal). The combination of HQ and HW was found in nine districts and the combination of HR and HW in four districts. Finally, when occurring, all combinations generally affected only 2–3 districts (AvgDistricts), which is comparable with the most common combination of HQ and strong wind gusts (3.8) (Table S5, above diagonal).

**Fig. 4:**
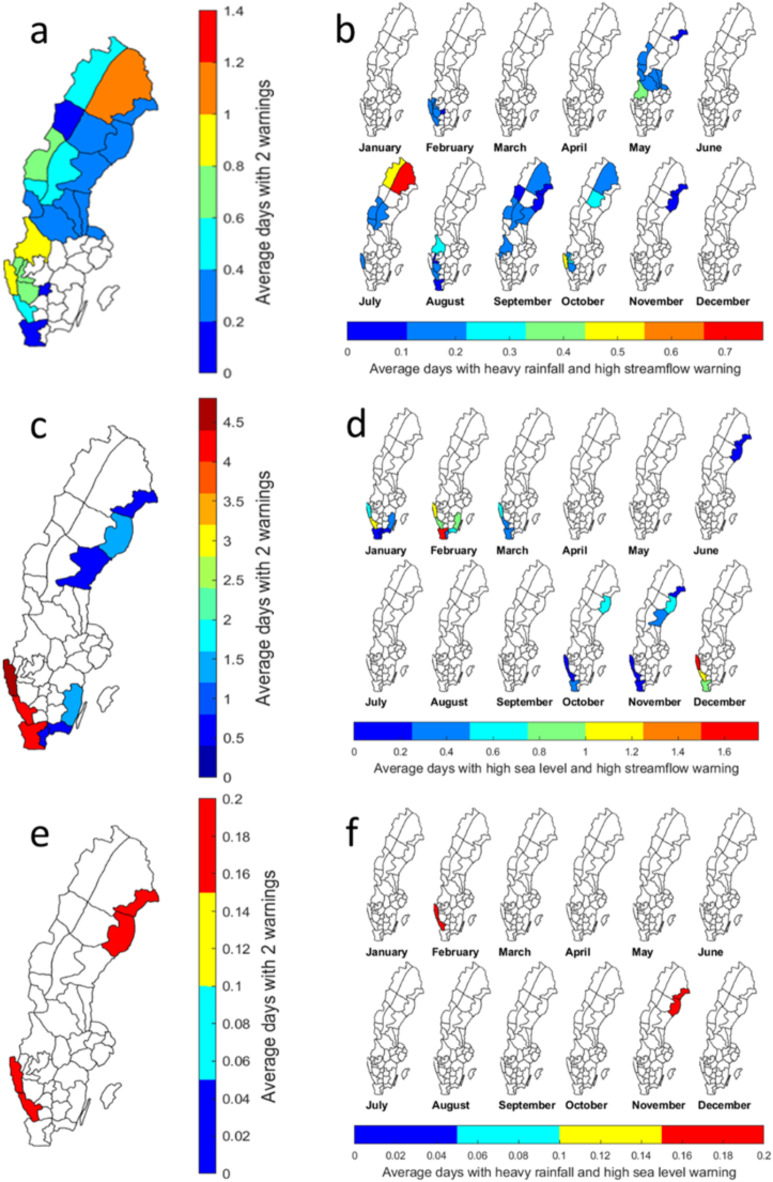
Average number of days per year and per month during 2011–2020 with warnings issued for the combinations of HR and HQ (**a**,** b**), HQ and HW (**c**,** d**), and HR and HW (**e**,** f**). Note the different scales of the colour bars. The maps were generated using the Mapping Toolbox in Matlab version 2018b (https://www.mathworks.com/products/mapping.html).

Concerning the spatio-temporal pattern of the issued combined flood-related warnings, an overview is shown in Fig. 4. When taking the seasonality of HR and HQ warnings into account (Fig. 3), the districts with elevated risk are mainly located in Norrbotten (WD2 in Fig. 1) and in southwest Sweden (WD16 and WD24) (Fig. 4a). Figure 4b shows that the potential risk of this combination has a low frequency, i.e., < 1 day per month in the 10-year period, mainly in the period May to October. In Norrbotten July stands out (Fig. 4b), which is around the end of the spring flood period and also in the season of high-intensity rainfalls. In the southwest, two combinations of area and month stand out: (*i*) Västra Götaland, Bohuslän and Gothenburg (WD24) in October, and (*ii*) Värmland (WD16) in May (Fig. 4b). It is primarily connected to the risk of HR in the summer half year, and sometimes follow a long and wet period or sometimes co-occur with the melting of early or late snow.

HW extremes, particularly in the Baltic Sea, can be considered as preconditioned events as storms frequently hit the basin when the mean sea level is already elevated^30^. As shown in Fig. 4c, HQ and HW often co-occurred in the southwest coastal districts (Fig. 4c). The southern-most district, Skåne, excluding Österlen (WD38), emerges as the most endangered in February, followed by Västra Götaland, Bohuslän and Gothenburg (WD24) and Halland (WD35). Also, the districts along the southern and south-east coast, i.e., Kalmar, excluding Öland (WD31) were often exposed to HQ and HW warnings during the same month. For northern Sweden such as Norrbotten, Västerbotten and Västernorrland (WD3, WD6 and WD9) along the northeast coast, in November a large area may be affected by this combination (see Fig. 4d).

Comparing to the other combinations, the co-occurrences of HR and HW were very limited (see Table 1). The southwest coast and northeast coastal districts are two risk-prone areas at both the monthly and annual scales (Fig. 4e and f). Combinations of all three warnings (HR, HQ and HW) were issued at two occasions during the 10-year period; in 2020 it happened in: (i) districts along the southwest coast in February and (ii) districts along the northeast coast in November.

### Validation events

As discussed above, we consider weather warnings a meaningful proxy for actual compound events, from a risk assessment perspective, even though the warnings are not always followed by an actual corresponding hazardous event. Moreover, the analysis supports the identification of actual (or potential) representative compound events, which may be used for post-event analysis and/or further preparedness. As warnings were actually issued for different time horizons (from hours up to a couple of days), the identified events will have a distinct connection to societal preparedness and response, which is an advantage compared with finding compound events based on observations alone.

**Fig. 5:**
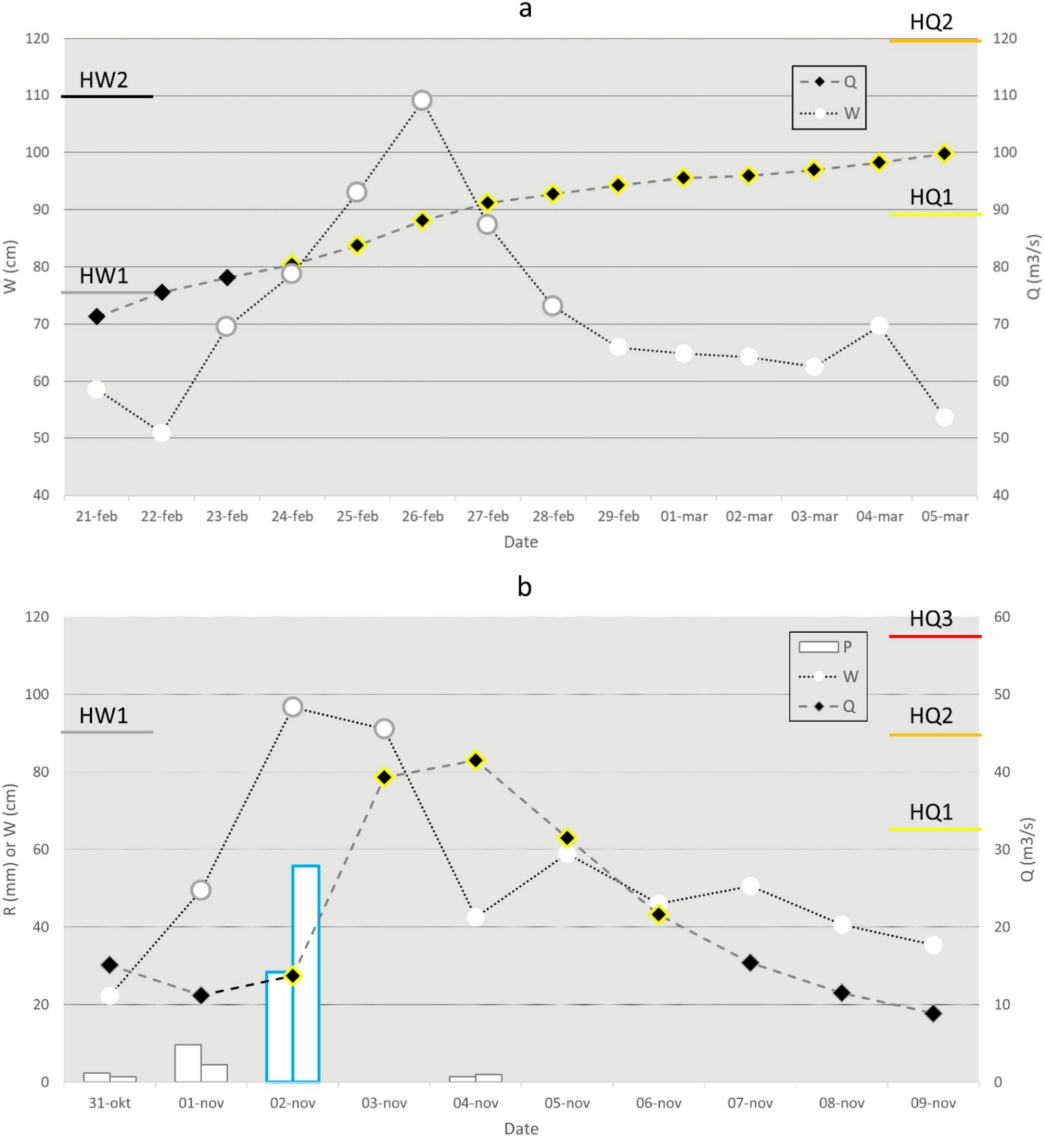
Two events with compound flood risk were identified from the analysis of warnings: at the northern east coast in February 2020 (**a**) and at the south-east coast in November 2020 (**b**). In both figures, observed daily streamflow (Q) and sea level (W) are shown as lines together with corresponding warning levels on the y-axes. In (b), also rainfall (P) at two stations in the basin are shown as bars. Coloured lines around the symbols or bar edges indicate that there was an active warning on that day.

Figure 5 shows two examples for compound events, where critical levels were reached for different flood-related hazards at the same time and in the same place. Both cases happened in 2020 and both are located along the Swedish east coast; one occurred in February in the south and one in November in the north (red dots in Fig. 1; see also Supplementary Information, Figures S2 and S3). The February 2020 event (Fig. 5a) was located at the mouth of River Emån, with a catchment area of ~ 4500 km². Following an extended period with precipitation on already saturated conditions, which resulted in HQ2 warnings for high streamflow in the upstream parts, the streamflow in the downstream part continually increased, and on 24 February, an HQ1 warning became active. The downstream flow continued to increase, reaching HQ1 level on 27 Feb, and remaining above the HQ1 level for almost one month. At the same time, repeated low-pressure systems led to elevated sea levels and on 23 February, an HW1 warning became active. The sea level peaked at almost HW2 level on 26 February, after which it decreased, and on 28 February the warning was removed. Flooding was reported at several places along the downstream reaches of River Emån.

The November 2020 event (Fig. 5b) was located at the mouth of River Dalkarlsån, with an area of 346 km². In the end of October, a low-pressure system with south-westerlies pushed the water in the Baltic Sea towards the north, rapidly increasing sea levels along the northern coast of Sweden. On 1 November an HW1 warning was issued and on both 2 and 3 November, the sea level remained above the HW1 level. At the same time, an HR1 warning was active, and on 2 November close to 60 mm of rainfall was observed in the river basin. A resulting HQ1 warning for high streamflow was issued already on the same day, and on 4 November the streamflow peaked close to HQ2 level. Severe flooding was reported in the area, with municipalities even advising citizens not to leave their house.

## Discussion

We have shown that analyzing archived weather warnings is a useful way to characterize compound natural hazards that are meaningfully connected to temporally and spatially overlapping warnings, specifically combinations of pluvial, fluvial and coastal flood risk. The intra-annual and intra-national patterns identified are conceivably relevant for planning of e.g. emergency resources on national, regional and local scales. The approach furthermore made it possible to identify actual compound events that may be useful for future in-depth studies. To work with actual warnings rather than e.g. arbitrary percentiles or threshold exceedances of different variables gives the study a clear connection to societal preparedness and response.

There are some limitations that should be emphasized. An obvious one is that issued (overlapping) warnings are not the same as actual (compound) events, where the former only implies a potential risk for the latter; some warnings are true, i.e. warnings levels are actually exceeded, and some do not lead to actual events. We have not been able to make a full investigation into the accuracy of the warnings, but since 2017 SMHI is obliged to present the accuracy of class-2 and -3 warnings in their annual report. Based on this reporting, the accuracy of class-2 and -3 warnings (i.e. the most challenging ones) in the period 2015–2020, as quantified by the Critical Success Index (CSI), is in the 60–70% range^31^, which gives an indication of the relationship between the most severe warnings and the actual outcome. A further indication of the accuracy is provided by the “forecast index”, an integrated metric reflecting the overall precision of the forecasts upon which the different warnings are made. On average, and over the full period 2011–2020 this metric is 83.6% for day-1 forecasts and 71.4 for day-5 forecasts, with little variation between years (for more information, see^31^). Overall, we believe that the accuracy of warnings and forecasts well represents a state-of-the-art weather forecasting system.

Another noteworthy limitation is the transformation of the warnings to a temporal scale of one day and a spatial scale of warning district. This simplification was required to make the analysis attainable within the resources given. In reality, some warnings cover only part of the day and/or part of the district, and therefore exact space-time overlaps may not always have been the case. However, it may be argued that from the perspective of first responders and others needing to react to warnings, exact overlap is of less importance but that multiple warnings on the same day and in the same district is always challenging. A final limitation is the length of the study period, 10 years, with only a few cases of some warnings and warning combinations.

Concerning compound flood risk in Sweden, the study indicates that the main challenge is the risk of combined fluvial and coastal flooding caused by an interaction of HQ with HW. This combination of warnings occurs on average almost five days per year along the southwest coast, primarily during the winter half year. Also, the east coast is at risk, but to a lesser degree. Overlapping warnings of HR and HQ, potentially leading to combined fluvio-pluvial flooding, occur primarily during the summer half year. The combination is found in almost the entire country except for the south-east part, with slightly elevated frequency in the south-east and in the very north. Overlapping warnings of HR and HW are very unusual.

We encourage similar investigations to be carried out in other countries, although we are aware of several potential complications. First of all, all issued warnings need to have been systematically saved along with all relevant meta data in terms of time, location, etc., during an extended period. This requires a dedicated and automatized system. Secondly, the procedures for issuing warnings should preferably have been stable and essentially unchanged throughout the period, to ensure consistency. This is a challenge as procedures regularly change in response to e.g. new opportunities provided by forecasting models and new demands from society. Finally, it is common that different warnings are issued by different authorities or institutes, conceivably with different systems, which may make it virtually impossible to compile a consistent data base. We therefore close by encouraging NMHSs and others that issue weather warnings to systematically save their warnings in order to identify spatio-temporal hot spots, investigate trends and generally provide society with the best possible support.

## Methods

### Meteorological, hydrological and oceanographical warnings

Warnings are centred on modelling data simulated by meteorological, hydrological and oceanographical models at SMHI and stored in the internal data base, KEPS. The database is designed for automatic storage of all types of warnings for 40 land districts and neighbouring water bodies (see Fig. 1). The warnings include heavy rainfall, heavy snowfall, high temperature, high streamflow, high sea level, strong wind gusts, high lake level and thunder strikes. In the KEPS-client, the issued warnings are displayed on a map where the affected districts are highlighted together with descriptive text including spatial and temporal information, start and end time of the warning, warning type and severity. Table S2 in Supplementary Information summarizes SMHI’s criteria for issuing warnings, as well as relevant potential consequences in which class 1 is the lowest severity level and class 2 or class 3 the highest.

### Meteorological forecasts

A meso-scale analysis system, MESAN, operationally run at an hourly timestep at SMHI, assimilates all available observations of meteorological variables from manual observations, automatic station data, satellite and radar imagery to an analysis. Together with meteorological deterministic and ensemble forecasts from European Centre for Medium-Range Weather Forecasts (ECMWF) as well as from other forecast centres, the analysis and forecasts are produced, taking into account the quality and representation of relevant observations. The forecasts are produced 7 days ahead primarily based on previous forecasts and a selected deterministic 10-day forecast released by ECMWF at 9 km resolution, and further downscaled by SMHI’s forecast model, AROME, to 2.5 km resolution for the coming 48 h.

### Hydrological (streamflow) forecasts

HQ forecasts, based on a national set-up of the hydrological HBV model^32,33^, have been in operation in Sweden since the early 1970s. Being a semi-distributed rainfall-runoff model, the HBV model is driven by precipitation and temperature, with numerical descriptions of hydrological processes at the basin scale. The model consists of routines for snow melt, soil moisture, groundwater discharge and routing through rivers and lakes. Basins with considerable elevation ranges can be subdivided into elevation zones which, if needed, can be further divided into different vegetation zones (e.g., forested and non-forested areas). These subdivisions are made for the snow and soil moisture routines only.

HBV-simulated daily mean streamflow in 10-day forecasts, together with hydrological forecasters’ expert knowledge, are used as a basis for issuing warnings with consideration of forecast reliability and lead time. The model’s forcing data, i.e. daily precipitation and temperature, are taken directly from the above meteorological forecast. The model’s initial conditions are updated to the forecast date with observations using weighted stations or gridded precipitation and temperature, if possible^34^.

### Oceanographical (sea level) forecasts

The oceanographical model NEMO is run at SMHI to forecast e.g. sea level, salinity and sea water temperature. The model is originally developed by a European consortium, adapted to the Baltic Sea by SMHI and termed NEMO-Nordic (www.nemo.ocean.eu). NEMO-Nordic simulates the entire area of the Baltic Sea, including water bodies along the southwest coast of Sweden (i.e., Kattegat and Skagerrak, Öresund and part of the North Sea). For short-range forecasts up to 2.5 days, the meteorological model HARMONIE-AROME is used to provide meteorological boundary conditions, while for longer-range forecasts, forcing data is taken from a global model at ECMWF.

There are two versions of NEMO-Nordic for forecasts, NSBS01 for short-range forecasts and NS02 for long-range forecasts. The NSBS01 NEMO-Nordic is run 4 times per day to make a short-range forecast at 1.85 km resolution for the coming 60 h, while the NS02 NEMO-Nordic is run 2 times per day to make a 10-day forecast at the horizontal resolution of 3.4 km.

### Binary indicator

To facilitate analysis, individual warnings were registered on a *daily* and individual *district* level, using a binary approach. That is, a day is registered as an “active warning day” for a certain warning district, when a type of warning is active at least once per day. The same definition is applied to a certain day, where a district is registered as an “active warning district” with at least a part of a warning district affected by a certain type of warning.

In mathematical notation, *S*_*tc*_*(i*,* j)* denotes the complete set of daily binary matrices for a certain warning with *t* representing the warning type (e.g. HQ) and c its corresponding warning class *c* (e.g. 2) for day *i* (1 ≤ *i* ≤ 3653; 10 years of data 2011–2020) and district *j* (1 ≤ *j* ≤ 40). Thus, for a warning type *t* and class *c*, *S*_*tc*_*(i*,* j)* = 1 when there is at least one “true” occurrence sometime during day *i* and somewhere in district *j*, otherwise *S*_*tc*_*(i*,* j)* = 0.

**Fig. 6:**
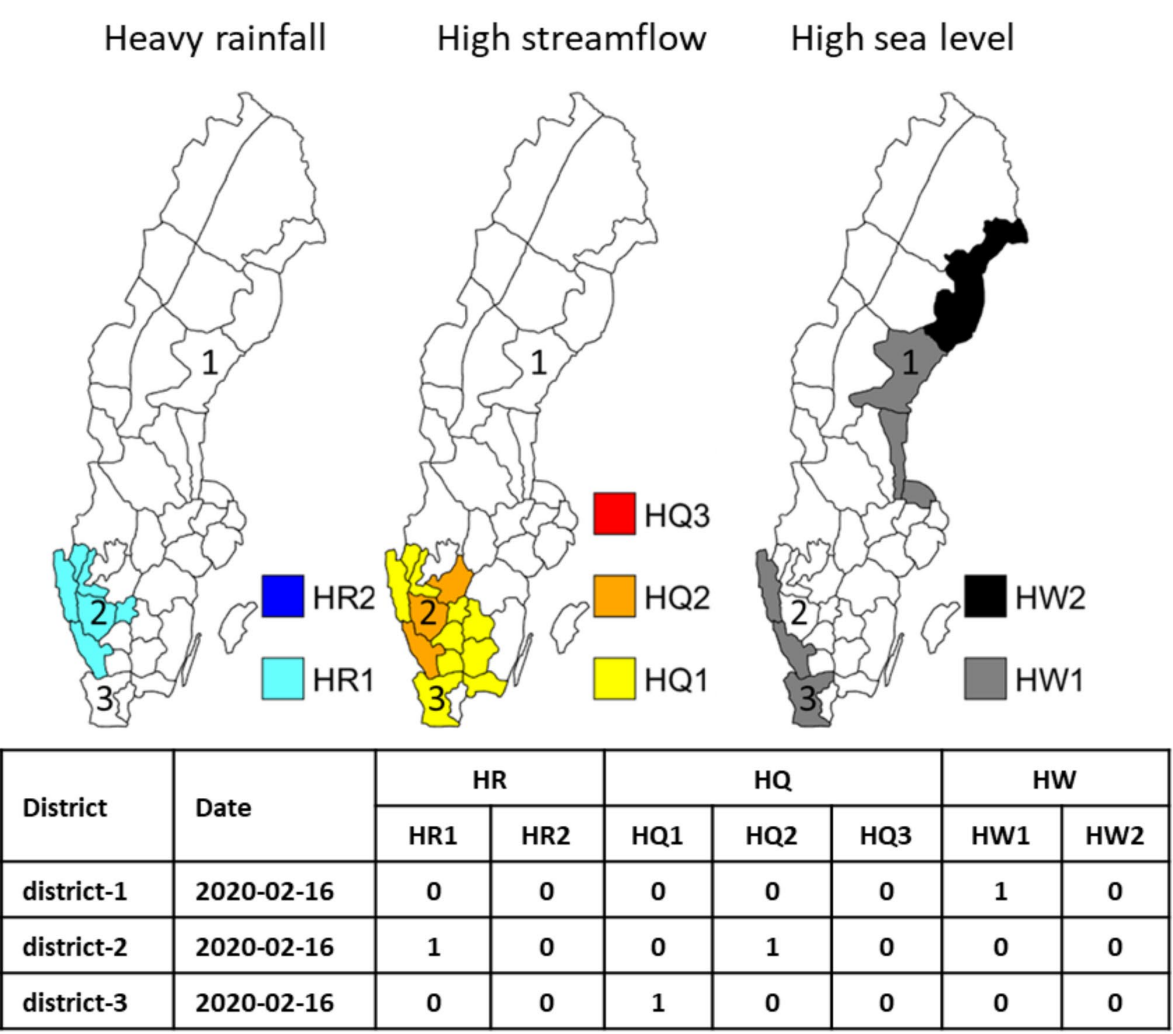
An example of a daily map of issued warnings (16 February, 2020) with corresponding values of S_tc_(i, j) in the table. The maps were generated using the Mapping Toolbox in Matlab version 2018b (https://www.mathworks.com/products/mapping.html).

The warnings issued on 16 February, 2020, are shown as an example illustrated in Fig. 6, where three types of warnings (i.e., HR, HQ and HW) were issued for different parts of Sweden and with a number of overlapping affected areas. The districts along the southwest coast were shown to be endangered by both HR and HQ, whereas HW warnings affected the northeast and southwest of Sweden. Here, different types of warnings are transformed to be a binary matrix, *S*_*tc*_*(i*,* j)*, for calculation during the analysis period, 2011–2020; for instance, an HQ2 warning in district 2 is represented by “1” in the corresponding columns.

The same type of binary matrix was used to characterize overlapping warnings. In this case, we do not distinguish between different classes, but all combinations of two warning types t1 and t2 are equivalent. The binary matrix with overlapping warnings will thus be denoted *S*_*t1t2*_*(i*,* j)*. If t1 is HR and t2 is HQ, for the case in Fig. 6, *S*_*t1t2*_*(i*,* j)* will thus be 0 for district 1, and 1 for district 2.

### Evaluation measures

We designed four different and complementary metrics – NumDD, NumDays, NumDistricts and AvgDistricts—to characterize and quantify the occurrences of individual warnings, as well as overlapping warnings (Table 2). Here, an overlapping warning can be any combination in which two (or more) types of warnings (of any class) are issued on part of the same day and in part of the same warning district. The calculated statistics were summed and averaged over calendar months and years. This allows us to identify (*i*) inter- and intra-annual variations of individual and/or overlapping warnings, (*ii*) specific years with the lowest and highest frequency of warnings, and (*iii*) hot spot districts affected by warnings.

**Table 2:** Definition of metrics: NumDD, NumDays, numdistricts and avgdistricts.

Metric	Definition	Question to answer	Mathematical expression	
			Individual	Overlapping
NumDD_**tc**_ NumDD_**t1t2**_	Total number of a specific warning or warning combination over all days *i* and all districts *j*	How common was this warning (combination) overall?	$\:{\sum\:}_{j=1}^{40}{\sum\:}_{i=1}^{3653}{S}_{tc}(i,j)$	$\:{\sum\:}_{j=1}^{40}{\sum\:}_{i=1}^{3653}{S}_{t1t2}(i,j)$
NumDays_**tc**_ NumDays_**t1t2**_	Number of days when a specific warning or warning combination occurred in at least one district *j*	How often did this warning (combination) occur anywhere in Sweden?	$\:{\sum\:}_{i=1}^{3653}{S}_{tc}\left(i,j\right)|j\ge\:1$	$\:{\sum\:}_{i=1}^{3653}{S}_{t1t2}\left(i,j\right)|j\ge\:1$
NumDistricts_**tc**_ NumDistricts_**t1t2**_	Number of districts where a specific warning or warning combination occurred on at least one day *i*	How many districts experienced this warning (combination)?	$\:{\sum\:}_{j=1}^{40}{S}_{tc}\left(i,j\right)|i\ge\:1$	$\:{\sum\:}_{j=1}^{40}{S}_{t1t2}\left(i,j\right)|i\ge\:1$
AvgDistricts_**tc**_ AvgDistricts_**t1t2**_	Average number of affected districts on days when a specific warning or warning combination occurred	When this warning (combination) occurred, how many districts were typically affected?	NumDD_tc_/NumDays_tc_	NumDD_t1t2_/NumDays_t1t2_

Note that they are used to quantify the occurrences of both individual warnings (of type t and class c) and overlapping warnings (of types t1 and t2).

## Electronic supplementary material

Below is the link to the electronic supplementary material.

Supplementary Material 1

## Data Availability

The dataset generated and analysed during the current study is not publicly available due to internal restrictions but are potentially available from the corresponding author on reasonable request.
