# Supplementary material for: Weather warning archives reveal spatio-temporal hot spots of compound natural hazards
Source: Sci Rep. 2025 Apr 17;15:13330. doi: 10.1038/s41598-025-96842-6 (PMC12006412; doi:10.1038/s41598-025-96842-6)
Supplement: Supplementary file 1 — Supplementary Material 1 [file 41598_2025_96842_MOESM1_ESM.pdf]

Supplementary Information

Table S1: The names of Swedish warning districts and waterbodies.

AL	Water bodies	Nr.	Land districts (County)	Nr.	Land districts (County)
A	Gulf of Bothnia	1	Norrbotten, north Lapland mountains	21	Södermanland
B	North Quark	2	Norrbotten, inland parts	22	Stockholm, excluding Roslagen
C	North Bothnian Sea	3	Norrbotten, coastal area	23	Västra Götaland, inner Dalsland
D	South Bothnian Sea	4	Västerbotten, the south Lapland Mountains	24	Västra Götaland, Bohuslän and Gothenburg
E	Åland Sea	5	Västerbotten, inland parts	25	Västra Götaland, south-west of lake Vänern
F	North Baltic Sea	6	Västerbotten, coastal area	26	Västra Götaland, north Västergötland
G	Middle Baltic Sea	7	Jämtland, the Jämtland mountains	27	Östergötland
H	South Baltic Sea	8	Jämtland, excluding the Jämtland mountains	28	Västra Götaland, Sjuhäradsbygden and Göta river
I	South-west Baltic Sea	9	Västernorrland	29	Jönköping, south of Lake Vättern
J	Öresund	10	Jämtland, the Härjedal mountains	30	Jönköping, east part
K	Kattegat	11	Dalarna, the Dala mountains	31	Kalmar, excluding Öland
L	Skagerrak	12	Gävleborg, inland parts	32	Jönköping, west part excluding south of Lake Vättern
		13	Gävleborg, coastal area	33	Kalmar, Öland
		14	Dalarna County, excluding the Dala mountains	34	Gotland
		15	Uppsala County, coastal area	35	Halland
		16	Värmland	36	Kronoberg, west part
		17	Örebro	37	Kronoberg, east part
		18	Västmanland	38	Skåne, excluding Österlen
		19	Uppsala, inland parts	39	Skåne, Österlen
		20	Stockholm, Roslagen	40	Blekinge

Table S2: Warning level of heavy rainfall, high streamflow and high sea level used at SMHI until October, 2021 ([1]Skagerack, Kattegatt, [2]Öresund, Södra Östersjön, and Bottenviken, [3]Mellersta Östersjön, Norra Östersjön, Ålands hav, [4]Södra Bottenhavet, Norra Bottenhavet, Norra Kvarken).

	Criteria	Consequences
**Heavy rainfall**	Class 1: Rainfall from 35 mm/12 hours - 70 mm/24 hours.	Risk of large water bodies; may cause overflowing stormwater pipes and flooded basements.
	Class 2: Rainfall more than 70 mm/24 hours.	Very high risk of flooding in basements, storm-water systems and smaller watercourses. Risk of flooded roads.
**High streamflow**	Class 1: High streamflow in watercourses at a level that occurs on average between every 5 - 25 years.	May cause minor flood problems.
	Class 2: Very high streamflow in watercourses at a level that occurs on average between every 25 - 50 years.	Flood problems in exposed areas.
	Class 3: Extremely high streamflow in watercourses at a level that occurs on average every 50 years.	Causes serious flood problems.
**High sea level**	Class 1: [1] > 80 cm in RH2000*; [2] > 90 cm in RH2000*; [3] > 75cm in RH2000*; [4] > 70cm in RH2000*<br>Class 2: [1] > 120 cm in RH2000*; [2] > 130 cm in RH2000*; [3] > 110cm in RH2000*; [4] > 100cm in RH2000*	

* Sweden's 3rd national precision weighing system. The zero level in RH 2000, is defined by Normaal Amsterdams Peil (NAP), a point in Amsterdam that is used as the zero point in other European countries.

Table S3: NumDD, NumDays, NumDistricts and AvgDistricts on average in 2011-2020.

Warning	Level	NumDD	NumDays	NumDistricts	AvgDistricts
**High streamflow**	Class I	462.8	105.5	26.1	4.3
	Class II	77.4	24.1	8.7	2.6
	Class III	8.5	3.8	2.4	1.3
**Heavy rainfall**	Class I	76.8	18.1	25.1	4
	Class II	0.2	0.1	0.2	0.2
**Heavy snowfall**	Class I	422.7	71.2	37.4	6.1
	Class II	16.1	6.5	6.8	2.5
**Strong wind gust**	Class I	208.7	37.1	30.1	5.6
	Class II	42.1	9.5	12	4.5
	Class III	2.2	0.7	1.1	0.5
**High lake level**	Class I	10.3	5.3	1	0.9
**High sea level**	Class I	185.5	43.6	13.5	4
	Class II	14.3	4.7	3.9	2.1
**Thunder**	Class I	43.9	7	15.5	4.5
	Class II	0.7	0.5	0.4	0.4
**High temperature**	Class I	49.6	5.9	11.4	5.7
	Class II	24.1	3.1	3.9	2.8

Table S4: A summary of the overlap between all warnings during 2011-2020. Above diagonal: NumDD, i.e. the total number of occurrences of this combination. Below diagonal: NumDays, i.e. the total number of days when this combination occurs in at least one district.

	Heavy rainfall	Heavy snowfall	High temp.	High stream-flow	High sea level	Strong wind gust	High lake level	Thun-der	
**Heavy rainfall**		0	0	93	8	17	0	5	
**Heavy snowfall**	0		0	155	249	100	0	0	
**High temp.**	0			0	0	0	0	7	
**High streamflow**	39	65	0		158	177	0	1	NumDD
**High sea level**	4	108	0	80		390	72	0	
**Strong wind gust**	7	47	0	47	165		0	0	
**High lake level**	0	0	0	46	0	0		0	
**Thunder**	2	0	2	1	0	0	0		
	NumDays								

Table S5: A summary of the overlap between all warnings during 2011-2020. Above diagonal: NumDistricts, i.e. the total number of districts having this combination at least once in the period. Below diagonal: AvgDistricts. i.e. the average number of districts when the combination occurs.

	Heavy rainfall	Heavy snowfall	High temp.	High stream-flow	High sea level	Strong wind gust	High lake level	Thun-der	
**Heavy rainfall**		0	0	23	4	9	8	5	
**Heavy snowfall**	0			26	16	31	0	0	
**High temp.**	0	0		0	0	0	0	7	
**High streamflow**	2.4	2.4	0		9	24	0	1	NumDistricts
**High sea level**	2	2.3	0	2		16	0	0	
**Strong wind gust**	2.4	2.1	0	3.8	2.4		0	0	
**High lake level**	0	0	0	1.6	0	0		0	
**Thunder**	2.5	0	3.5	1	0	0	0		
	AvgDistricts								

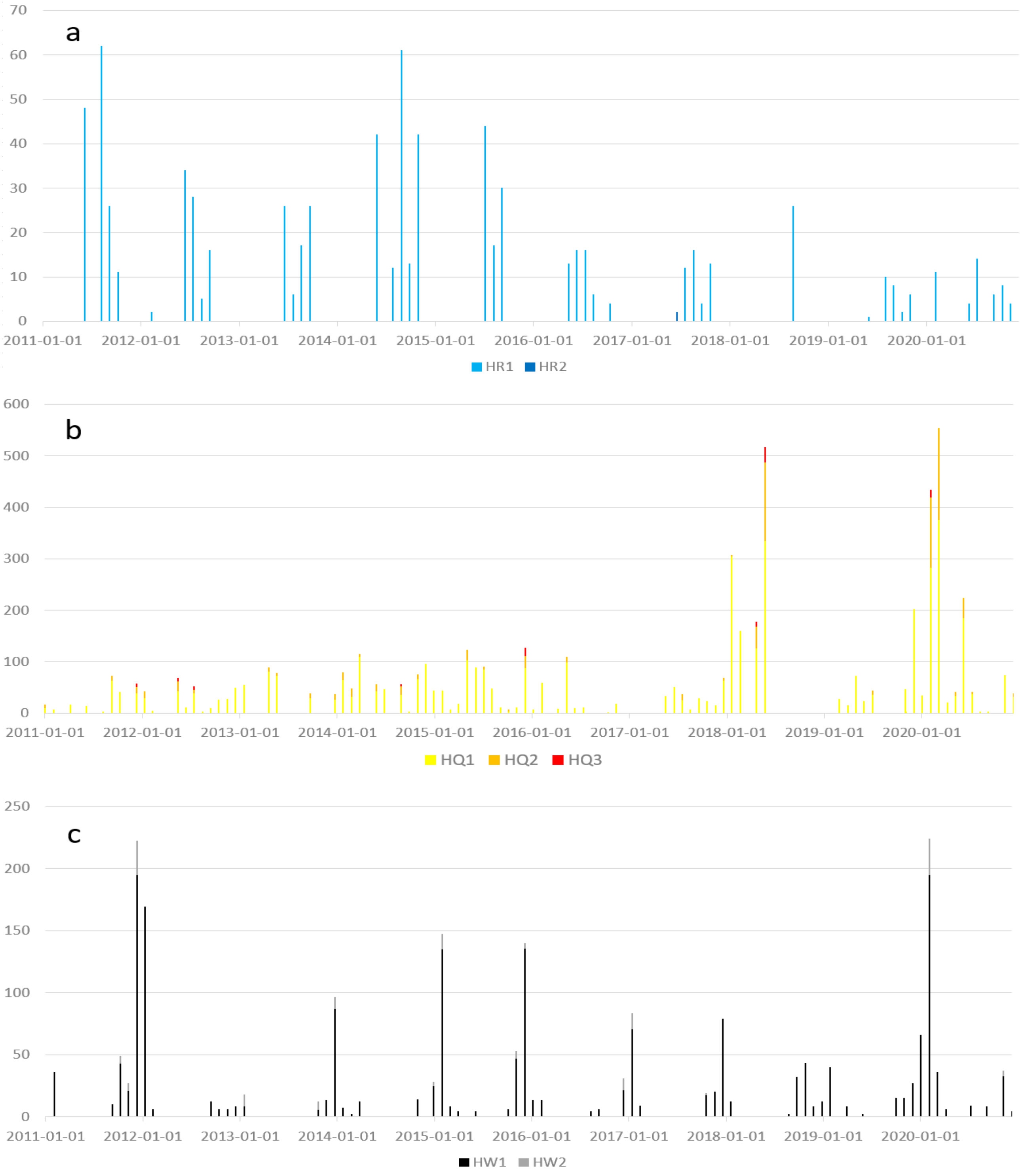

Figure S1: Flood-related warnings of heavy rainfall (a), high streamflow (b) and high sea level (c), summed over warning districts per month in Sweden from 2011 to 2020.

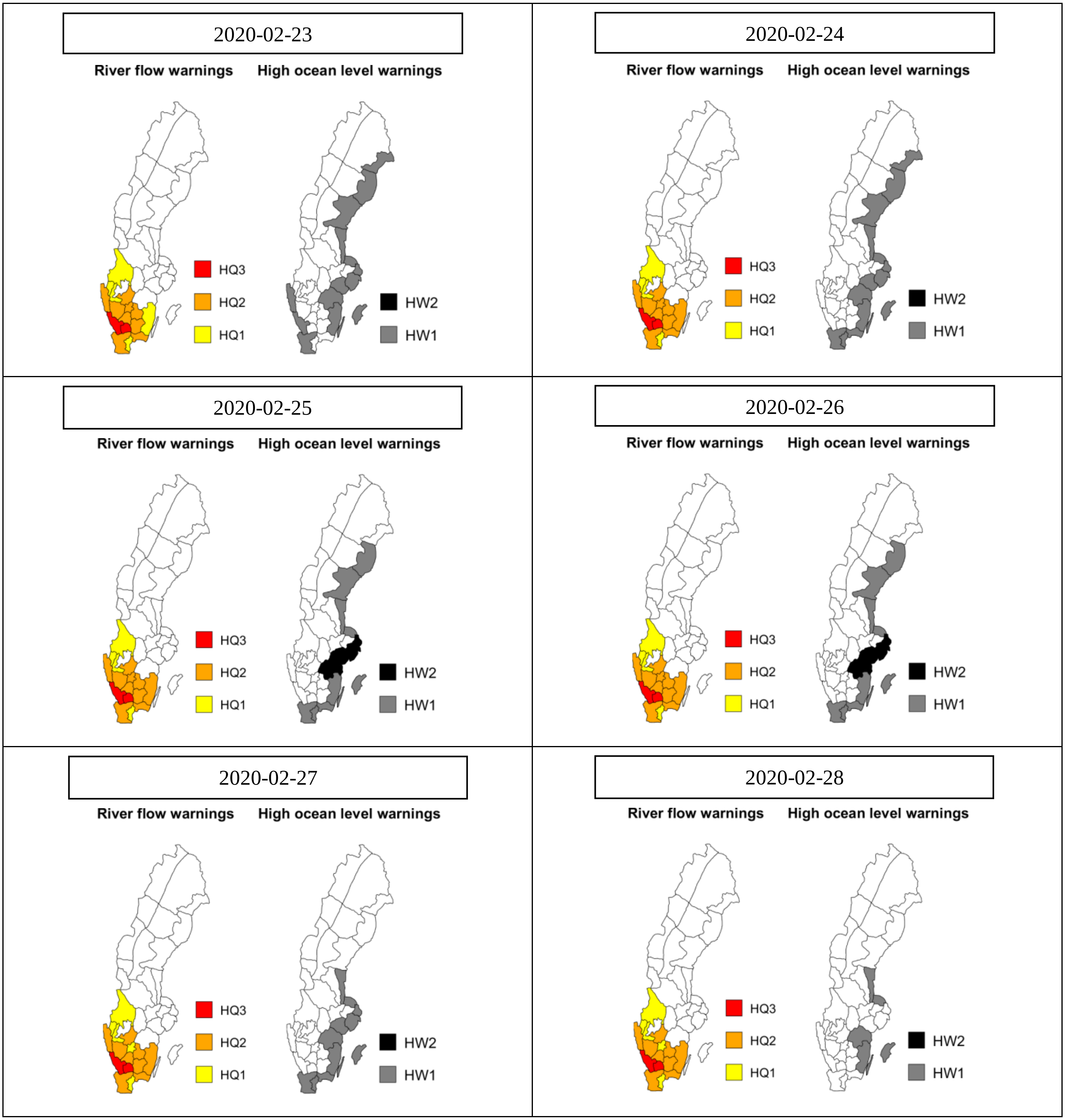

Figure S2: A compound flood event including high streamflow (HQ) and high sea level (HW) in February 2020. The maps were generated using the Mapping Toolbox in Matlab version 2018b (https://www.mathworks.com/products/mapping.html).

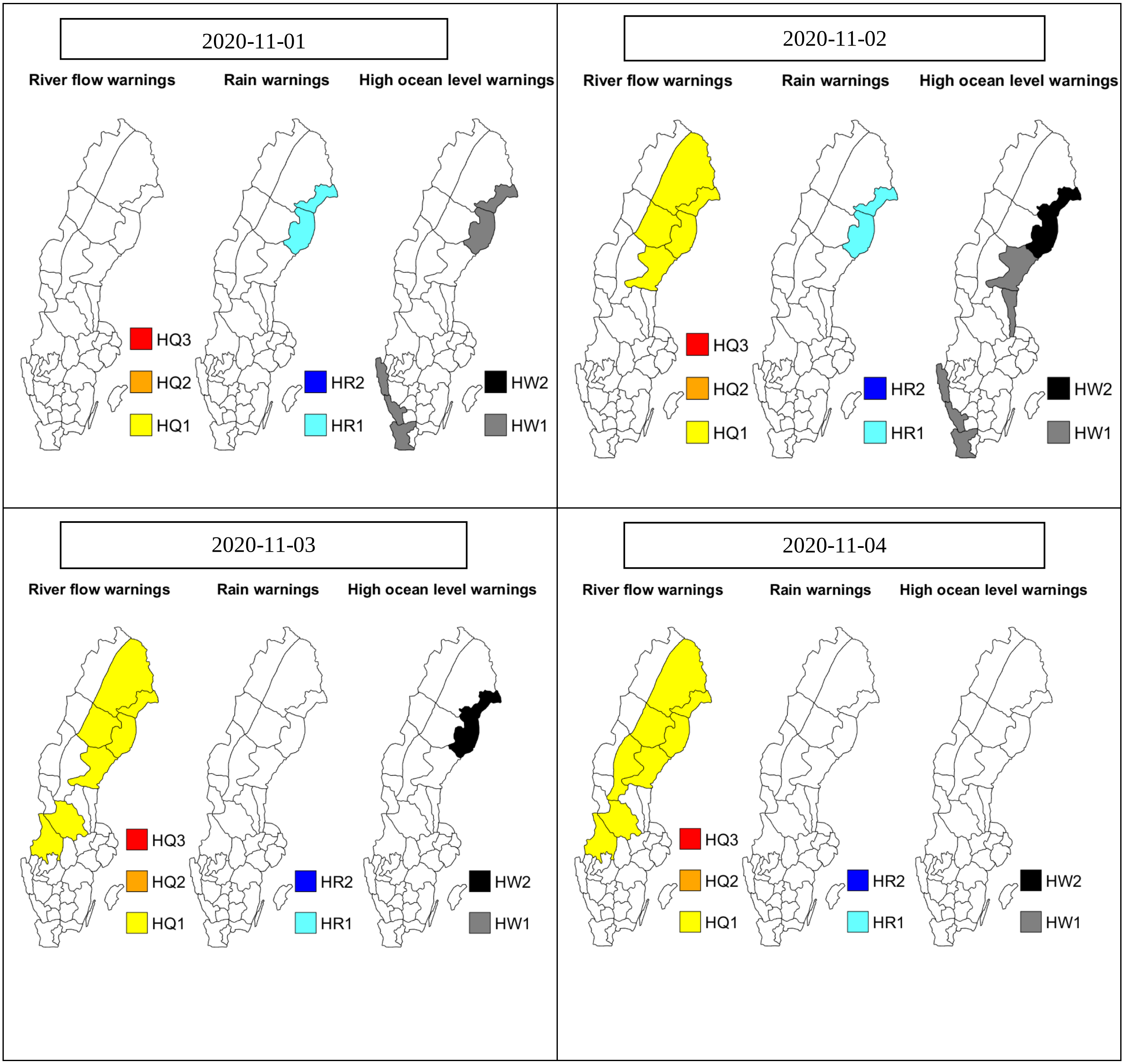

1 **Figure S3: A compound flood event including heavy rainfall (HR), high streamflow (HQ) and high sea level (HW) in November 2020.**
2 **The maps were generated using the Mapping Toolbox in Matlab version 2018b**
3 **(https://www.mathworks.com/products/mapping.html).**
